# The treatment of accidental hypothermia and drowning with the aid of extracorporeal circulation/ECMO

**DOI:** 10.1186/1749-8090-8-S1-O83

**Published:** 2013-09-11

**Authors:** K Visser, R Schepp

**Affiliations:** 1 Heart Centre Friesland, the Netherlands

## Background

The Heart Centre Friesland is the youngest cardiac surgery facility in the Netherlands, starting in January 2004. It is located in the northern part of the country serving 750000 inhabitants.

The environment is very water rich, with a lot of recreational activities on the water, in summer and winter time. Apart from that, there is an extensive off shore industry in our part of the North Sea.

The expectation was that a certain number of hypothermic patients, with or without drowning, should be presented to our centre. This was not the case initially. A survey in the region among rescue services and hospitals showed that approximately 20 patients per year existed. Obviously, the first line rescue teams were not aware of the proper way to handle this patient category. A lot of energy was spent to inform the first line how to treat the patients and when to go to a heart centre. The initial treatment is simple, intubate the patient, start resuscitation and measure the core temperature. If the temperature is 28 °C or lower, transport to a heart centre. Any degree of hypothermia with cardiac instability, the same action, and any case with fluid aspiration too.

The aim of this presentation is to show an overview of the patients we treated. One of these patients will be used as a case report to show the techniques used for treatment.

Seven patients were presented to our centre in the last two years. Four of the patients were severely hypothermic, three with mild hypothermia. Six of the patients were submerged and had aspirated large volumes of water.

## Methods

Three of the deep hypothermic patients where discharged without any morbidity, the last one was weaned from bypass, but suffered from cerebral damage due to insufficient resuscitation. All mild hypothermic patients could be weaned from bypass, but predictably suffered severe cerebral damage. One of them became a non beating donor patient after five days of ECMO. Several cannulation techniques were used, depending on logistic possibilities and the indication, cardiac or pulmonary support. In the cardiac group we used three times a heart lung machine and two times mobile device with femoral cannulation. For the pulmonary insufficiency patients the Cardiohelp with veno-venous cannulation was used. Bypass time ranged from 2 to 125 hours, depending on the recovery of the pulmonary function.

The fact that all patients can be weaned from bypass taught us that drowning is not necessarily a contra indication for treatment.

This case report is about a 31-year-old woman with severe hypothermia, who was presented at the OR of the Medical Centre Leeuwarden on December 10, 2010. She was found in her car under the ice, and was submersed for about 45 minutes, before the Mobile Medical Team arrived. Ventricular fibrillation occurred during intubation. Resuscitation was immediately started and continuously performed during the transport to the hospital. The patient arrived at the hospital while resuscitation was performed. At that moment her rectal temperature was 21°C and her peripheral temperature 14°C.

Fulminant pulmonary oedema was present, and the patient was in her 12^th^ week of pregnancy.

We prefer to treat these patients in an OR with median sternotomy using a heart lung machine, but in this case we were forced to use the catheterisation lab to percutanuosly cannulate the femoral artery and vein. The Cardiohelp system was used to restore circulation. The disadvantage of this technique is the fact that in case of pulmonary insufficiency, the system is not optimal for ECMO and decompression of the left ventricle is not possible with the risk of stunning.

She was immediately anaesthetized. Nasopharyngeal, rectal en peripheral temperature-probes were placed. The initial dose of heparin was 500 IU. If the ACT < 200 sec. and the fibrinogen > 1,5 gr/l, a maintenance infusion of 500 IU/hour is started. After 6 hours we work strictly with the APTT between 60-80, in combination with measurements of thrombocytes, fibrinogen, d-dimers and AT III.

Circulation was restored with a cardiac index of 2,4. The arterial blood temperature was maintained at 21 °C. This was to convert the patient biochemically from a severe Ph-stat situation to an alpha-stat acid base regulation. Only after this, the patient was carefully rewarmed, while correcting the metabolic part of the acidosis.

Rewarming to 34 °C rectal temperature took about 7 hours. The patient was not rewarmed further, to facilitate the resuscitation protocol on the intensive care. After defibrillation and rewarming, the patient could not be weaned because of severe ARDS due to aspiration. The second day of support, the pulmonary function recovered, but stunning of the myocardium forced us to continue circulatory support. Finally, after 60 hours of support, the patient could be weaned from bypass. Unfortunately, a compartment syndrome occurred because of thrombus on the arterial canula.

## Results

The baby was lost on bypass. After decanulation, the patient showed a severe neurological status for two days. But starting the third day, she slowly recovered and walked, shortly after Christmas, by herself to the general nursing ward. At this moment, she has a complete neurological recovery and can perform her work as lawyer without any problem.

She recently visited our centre, she was there for a medical check-up, not for this accident, but because she was pregnant again.

## Conclusion

Although the amount of patients is not very high, the fact that these patients are most of the time completely healthy and young before the incident rectifies a protocollised approach. Regionally, it is of the utmost importance to train and inform the rescue services. For a good outcome the primary decision and a good logistical chain are very important.

Intramural, a proper medical protocol is necessary, because the complexity of these procedures is too high to allow improvisation.

Especially deep hypothermic patients are not doomed, even in the presence of drowning.

The medical team should be prepared and motivated to continue the treatment with cardiac and/or pulmonary support for a longer period after the initial resuscitation.

